# Adult Presentation of a Large Congenital Pulmonary Airway Malformation Discovered After Traumatic Rib Fractures

**DOI:** 10.7759/cureus.116530

**Published:** 2026-09-19

**Authors:** Brenda Hranec, Luke Hudson, Matthew Khouzam, Jacob W Hensley, Daniel Layish, Nayer Khouzam

**Affiliations:** 1 College of Medicine, Florida State University, Tallahassee, USA; 2 Department of Cardiothoracic Surgery, Indiana University School of Medicine, Indianapolis, USA; 3 Department of Pulmonary Medicine, AdventHealth Orlando, Orlando, USA; 4 Department of Cardiothoracic Surgery, AdventHealth Orlando, Orlando, USA

**Keywords:** benign and malignant tumors, cardiothoracic and vascular surgery research, congenital cystic lung lesion, congenital pulmonary airway malformation (cpam), pulmonary lobectomy

## Abstract

Congenital pulmonary airway malformation (CPAM) is a rare developmental lung lesion that is usually identified prenatally or in early childhood, and adult presentations are uncommon. A 44-year-old woman presented after a fall with right-sided chest pain and dyspnea. CT chest revealed nondisplaced fractures of the right seventh through ninth ribs and incidentally identified a large multilocular cystic lesion in the right lower lobe consistent with CPAM, unchanged from imaging performed 15 years earlier. Pulmonary function testing demonstrated mild small-airway dysfunction, air trapping, and reduced diffusing capacity without restrictive physiology. Given the lesion’s size and potential risk for infection or malignant transformation, the patient underwent flexible bronchoscopy followed by right muscle-sparing thoracotomy with right lower lobectomy and thoracic lymphadenectomy. Intraoperative findings included dense vascular adhesions and distorted tissue planes. The postoperative course was uncomplicated. Pathology confirmed a benign cystic congenital pulmonary lesion without malignancy. This case highlights an uncommon adult presentation of long-standing CPAM incidental discovery and underscores that radiographic stability does not exclude clinically significant disease requiring surgical management.

## Introduction

Congenital pulmonary airway malformation (CPAM), historically referred to as congenital cystic adenomatoid malformation, is a rare developmental lung lesion characterized by abnormal airway branching and replacement of normal lung parenchyma by cystic or adenomatoid tissue. The terminology and expanded classification of CPAM were established to reflect the spectrum of cystic and noncystic developmental airway lesions, which are generally classified according to the presumed level of airway origin and histopathologic appearance [1]. CPAM is most commonly diagnosed prenatally or during infancy and childhood; however, some lesions remain undetected until adulthood, when they may be found incidentally or after respiratory complications [2,3].

Congenital cystic pulmonary lesions are identified in approximately 1 in 10,000-30,000 live births, and CPAM is detected on roughly 1 in 2,700 prenatal ultrasound examinations [1]. Adult CPAM cases are a small minority, and management remains challenging because evidence is limited and many recommendations are extrapolated from pediatric congenital lung malformation literature. In a systematic review of 980 adult patients with congenital lung malformations, Pederiva et al. reported that 74.4% of adults were symptomatic at diagnosis, while 25.6% were asymptomatic and diagnosed incidentally [2]. The authors also reported a mean age at diagnosis of 34 years. Among symptomatic adult patients with CPAM, pneumonia was the most frequent presentation (31.1%), followed by cough, hemoptysis, and pneumothorax, and 31.2% reported recurrent respiratory infections, with the right lower lobe being involved in one-third of cases [2]. Resection was performed in 94.9% of adult CPAM cases, most commonly lobectomy (79.4%), with surgical complications in 4.4% [2].

Surgical resection is often considered in adult CPAM because it provides definitive histopathologic diagnosis and may reduce the risk of recurrent infection, pneumothorax, hemoptysis, progressive parenchymal injury, and rare malignant transformation. In the same adult systematic review, CPAM was associated with lung tumors in 25.6% of reported adult CPAM cases, most commonly adenocarcinoma [2]. Although this rate may be influenced by publication bias in case-based literature, these concerns remain clinically relevant when evaluating large, persistent cystic lung lesions in adults. Surgical decision-making must also consider lesion size, location, symptoms, pulmonary reserve, and the feasibility of complete resection [4-6]. We describe an adult patient in whom a large, long-standing CPAM was discovered incidentally during evaluation for traumatic rib fractures and was managed with definitive lobar resection.

## Case presentation

A 44-year-old woman presented to the emergency department after falling from her bed, with right-sided rib pain radiating to the back and difficulty breathing. A right lower lobe cystic lesion had been documented on imaging in November 2011, approximately 15 years before presentation. The patient reported that she had not been informed of this finding, and no interval imaging or pulmonary follow-up had been performed. She denied a history of recurrent pneumonia, hemoptysis, or chronic respiratory symptoms during the intervening period. Her medical history was additionally notable for sickle cell trait, alpha thalassemia, a motor vehicle accident in 2006, and active cigarette use of approximately one pack per day for the past 20 years. An MRI three months earlier had shown cholelithiasis and splenomegaly. On presentation, she was found to have severe anemia, a thyroid nodule, vitamin D deficiency, and incomplete right bundle branch block. Family history was notable for lupus in her mother. The patient was admitted for observation. A timeline of relevant prior medical events and the current hospitalization is shown in Figure 1.

**Figure 1:**
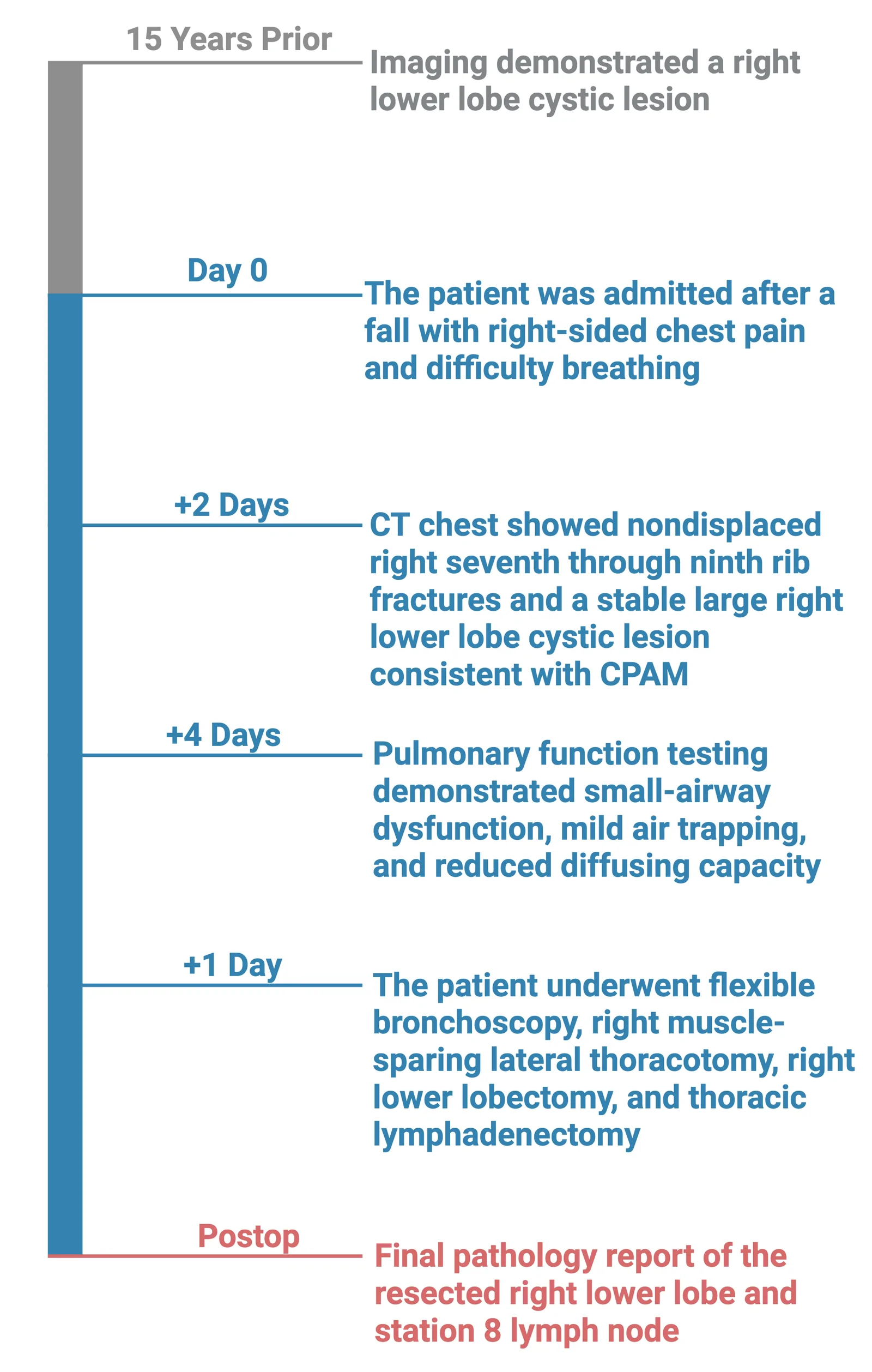
Timeline of the patient’s clinical course, diagnostic workup, and surgical management. CPAM, congenital pulmonary airway malformation This figure was created by the authors using BioRender (BioRender, Toronto, Canada).

CT chest without contrast (Figure 2) was obtained due to right-sided chest pain and difficulty breathing after the fall. Imaging demonstrated nondisplaced acute fractures of the right lateral seventh, eighth, and ninth ribs. There was no pneumothorax or pleural effusion. The scan also demonstrated a stable, complex, multilocular cystic lesion in the right lower lobe measuring 6.6 × 9.2 × 5.8 cm. The lesion contained abnormal adjacent parenchyma with an absence of pulmonary vessels, and the radiographic appearance was consistent with CPAM. This finding was stable compared with prior imaging from 2011 that measured the lesion at roughly the same size. Linear pleural-parenchymal scarring was also noted at the left lung base. Table 1 pulmonary function testing shows pulmonary dysfunction, air trapping, and a reduced diffusing capacity of the lungs for carbon monoxide.

**Figure 2:**
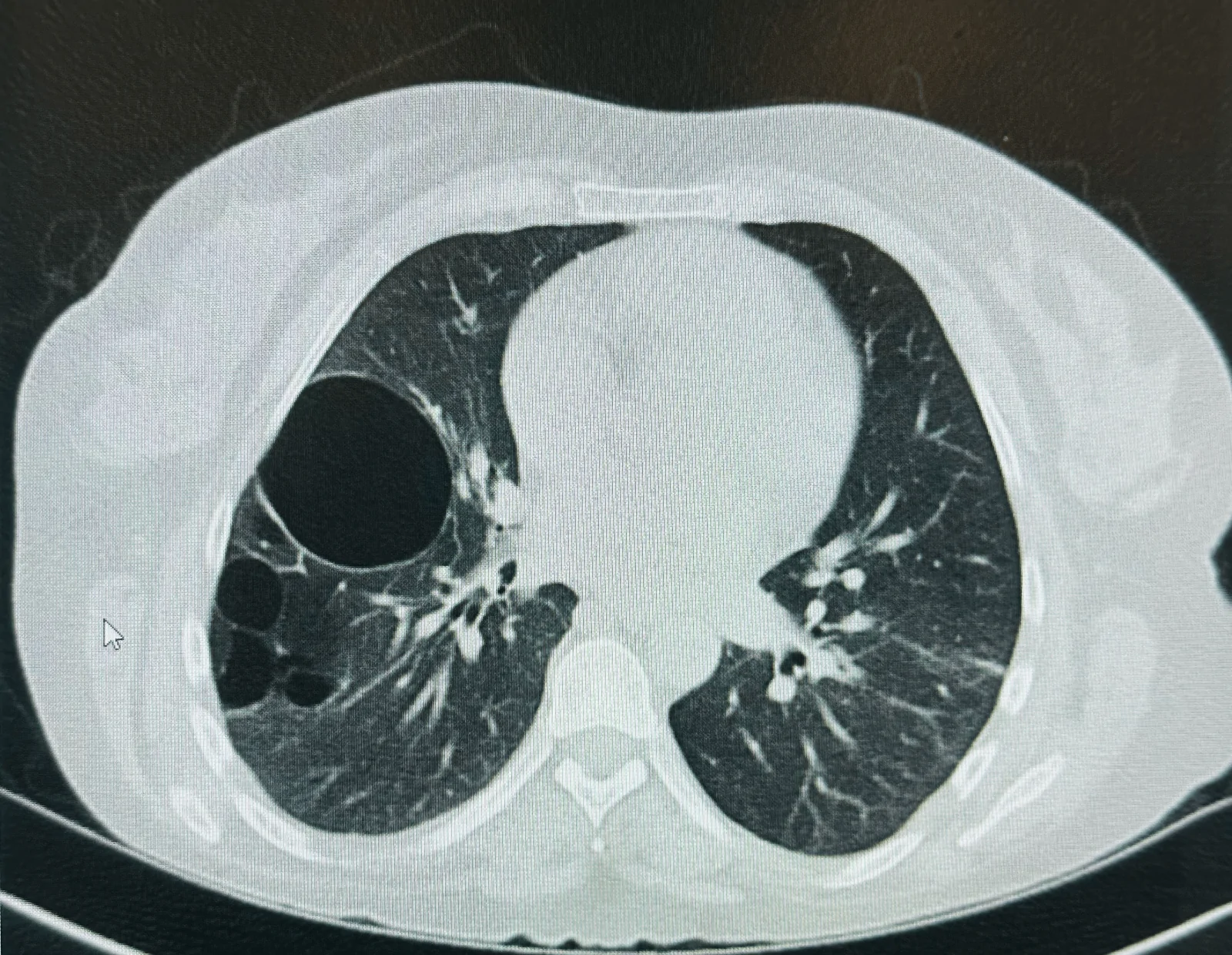
CT of the chest demonstrated a 6.6 × 9.2 × 5.8 cm complex, multilocular cystic lesion in the right lower lobe, consistent with CPAM. CPAM, congenital pulmonary airway malformation

**Table 1:** Preoperative pulmonary function testing demonstrating mild small-airway dysfunction, significant air trapping, and reduced diffusing capacity in the absence of restrictive physiology. DLCO = diffusing capacity of the lung for carbon monoxide; FEF₂₅–₇₅ = forced expiratory flow at 25–75% of FVC; FEV₁ = forced expiratory volume in 1 second; FEV₁/FVC = ratio of forced expiratory volume in 1 second to forced vital capacity; FVC = forced vital capacity; RV = residual volume; RV/TLC = residual volume-to-total lung capacity ratio; TLC = total lung capacity

Parameter	Measured	% Predicted
FVC	2.26 L	80
FEV₁	1.71 L	72
FEV₁/FVC	76%	91
FEF25-75	1.34 L/s	52
TLC	4.49 L	99
RV	2.14 L	179
RV/TLC	48%	182
DLCO	10.21 mL/min/mmHg	54
Corrected DLCO	12.96 mL/min/mmHg	68

Given the size of the lesion, its congenital appearance, lobar involvement, and concern for future complications, thoracic surgery evaluation was pursued. The patient underwent flexible bronchoscopy followed by right muscle-sparing lateral thoracotomy with right lower lobectomy and thoracic lymphadenectomy under general anesthesia.

Intraoperatively, extensive dense vascular adhesions were encountered throughout the operative field, with distorted tissue planes. Large cystic air spaces were present within the right lower lobe. The right lower lobe specimen was sent for pathologic examination. Estimated blood loss was 40 mL. Two right pleural chest tubes were placed. There were no intraoperative complications, and the patient tolerated the procedure well.

The surgical specimen consisted of a right lower lobectomy measuring 13.5 × 11.1 × 7.5 cm. A 6.0 × 3.9 cm collapsed, disrupted cystic airspace was identified along the medial and posterior aspects of the specimen, in close proximity to the stapled parenchymal resection margin, which was inked blue for orientation. The gross appearance of the resected lobe is shown in Figure 3.

**Figure 3:**
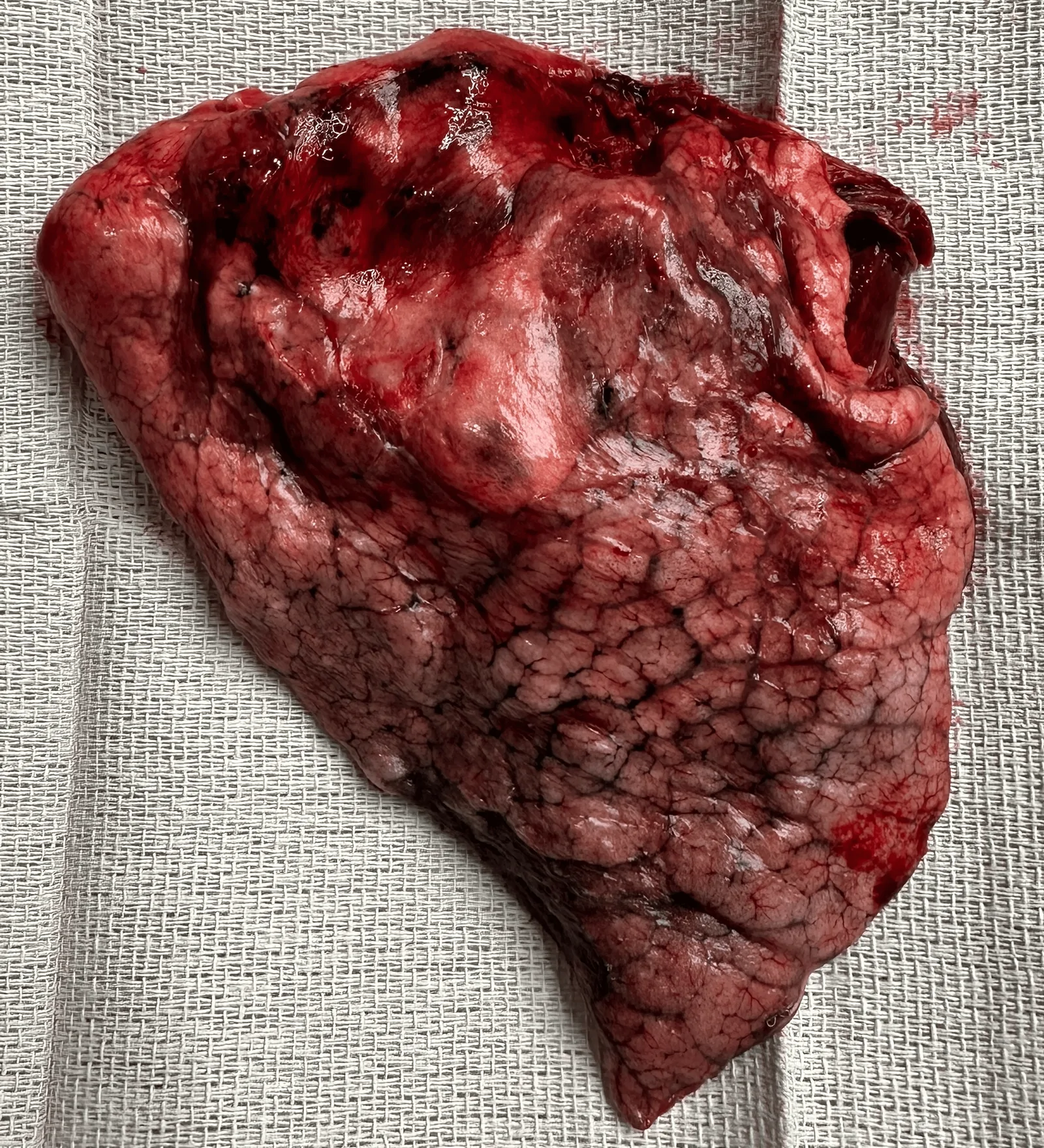
Intraoperative image of the resected right lower lobe specimen, demonstrating large cystic air spaces.

On gross examination, the bronchial lumen demonstrated a smooth tan lining without endobronchial lesions or obstruction. Serial sectioning revealed a markedly abnormal cystic and parenchymal architecture characterized by multiple enlarged and disrupted air-filled cystic spaces predominantly involving the superior, medial, and posterior regions of the lobe. No discrete nodules or masses were identified.

Microscopic examination demonstrated benign lung tissue characterized by multiple distorted cystic structures lined by bronchial epithelium, consistent with CPAM. Foci of elastosis were present. A mucicarmine stain showed no significant mucin production, arguing against a mucinous epithelial proliferation. There was no evidence of malignancy in the lobectomy specimen, and one station 11 lymph node was negative for malignancy. Photomicrographs of the histologic slides were not available for inclusion in this report.

Overall, gross and microscopic findings were consistent with a cystic congenital pulmonary lesion in the setting of CPAM, without evidence of an associated neoplasm. The patient demonstrated clinical improvement postoperatively with resolution of acute symptoms related to rib fractures and no new respiratory complications during the immediate recovery period. She was discharged in stable condition with appropriate follow-up arranged with thoracic surgery and pulmonology. This case demonstrates successful definitive surgical management of an incidentally discovered adult CPAM with an uncomplicated perioperative course and favorable short-term outcome.

## Discussion

Adult CPAM is rare because most lesions are diagnosed prenatally, during infancy, or in childhood. In adults, presentation is variable and may include recurrent pneumonia, cough, hemoptysis, pneumothorax, dyspnea, or incidental discovery during unrelated imaging. Smith et al. described asymptomatic adult CPAM as a rare presentation that creates management challenges regarding observation versus resection [3]. In the adult congenital lung malformation systematic review by Pederiva et al., 74.4% of patients were symptomatic at diagnosis, while 25.6% were asymptomatic and diagnosed incidentally [2]. Among adult CPAM patients, recurrent respiratory infections were reported in 31.2% of cases [2]. Our patient’s lesion was identified during imaging for an unrelated traumatic event, which is consistent with the subset of adult cases discovered incidentally.

The long-standing stability of the lesion in this patient is clinically important. While the absence of interval growth may suggest an indolent course, stability alone does not eliminate the risk of future complications. Ioachimescu and Mehta described a case of bronchioloalveolar carcinoma arising in an area of CPAM with a long period of apparent stability before later progression to invasive and metastatic adenocarcinoma [7]. The authors emphasized that lack of growth over many years cannot be fully relied upon as a marker of benignity [7]. This point is particularly relevant in the present case because the lesion had remained radiographically stable for approximately 15 years.

The decision to pursue surgery in this patient was based on diagnostic and preventive considerations. Although the lesion was not acutely infected at presentation, it was large, multiloculated, localized to the right lower lobe, and associated with abnormal surrounding parenchyma. Adult congenital lung malformations carry risks of recurrent infection, hemoptysis, pneumothorax, progressive parenchymal injury, and rare malignant transformation. In the systematic review by Pederiva et al., CPAM was associated with lung tumors in 25.6% of reported adult CPAM cases, with adenocarcinoma being the most common associated malignancy [2]. Although this rate should be interpreted cautiously because of publication bias, it supports careful evaluation and histopathologic confirmation in adult patients with persistent cystic lung lesions.

The role of surgery in asymptomatic or incidentally discovered CPAM remains debated, particularly because much of the literature focuses on pediatric patients. Kapralik et al. performed a systematic review and meta-analysis comparing surgical and conservative management of CPAM in children and highlighted the lack of consensus regarding optimal management of asymptomatic lesions [4]. In adult patients, the uncertainty is even greater because prospective adult data are limited. However, adult lesions have had more time to develop recurrent inflammation, infection, structural distortion, or occult premalignant or malignant change. For this reason, management should be individualized through multidisciplinary discussion, balancing operative risk against lesion size, symptoms, imaging features, pulmonary reserve, and patient preference.

The choice of formal right lower lobectomy rather than limited resection was also appropriate. In congenital pulmonary malformations, the extent of resection depends on lesion size, location, involvement of adjacent parenchyma, and feasibility of complete removal. While wedge resection or segmentectomy may be considered for small peripheral lesions, this patient’s lesion measured more than 9 cm in greatest dimension and involved the right lower lobe with abnormal adjacent tissue. Limited resection could have risked incomplete removal of abnormal congenital lung tissue. Bhende et al. noted that lobectomy has conventionally been preferred for CPAM localized to a single lobe, although lung-sparing resections are increasingly discussed in selected cases [6]. In a multicenter study comparing lobectomy with sublobar resection for congenital pulmonary malformations, Marinucci et al. found a lower rate of symptom recurrence after lobectomy compared with sublobar resection and concluded that lobectomy may provide more stable symptom remission when complete removal is required [5].

Intraoperative findings further supported the complexity of adult CPAM resection. The operative field demonstrated extensive dense vascular adhesions, distorted tissue planes, and large cystic air spaces within the right lower lobe. These findings suggest that adult CPAM may be technically challenging even in the absence of active infection at presentation, possibly reflecting years of chronic inflammation, prior subclinical infection, or tissue remodeling. Despite these challenges, the patient tolerated the operation well, with low estimated blood loss and no intraoperative complications.

This report is limited by its single-patient design and short postoperative follow-up. Tobacco use and underlying hematologic conditions, including severe anemia, may have contributed to the abnormal pulmonary function findings, limiting attribution to CPAM. Finally, the 2011 imaging was unavailable for direct review, so lesion stability was based on the radiology report.

This case is notable for several reasons. First, the lesion was large and lobar, measuring more than 9 cm in greatest dimension. Second, it remained radiographically stable for approximately 15 years, highlighting that long-term stability does not necessarily eliminate clinical significance. Third, the lesion was discovered during evaluation for an unrelated traumatic event, illustrating how adult CPAM may remain silent for years. Finally, the case supports the role of multidisciplinary evaluation and definitive surgical resection for large congenital cystic lung lesions in adults.

## Conclusions

Adult CPAM is rare and may be discovered incidentally during imaging performed for unrelated symptoms or injuries, as occurred in this patient during evaluation of traumatic rib fractures. Long-term radiographic stability does not fully exclude future clinical significance or malignant potential, and a lesion that has been unchanged for many years may still warrant definitive management. In this case, surgical resection provided a definitive histopathologic diagnosis, removed abnormal congenital lung tissue, and addressed the risk of future complications. For large, lobar, multiloculated lesions or lesions associated with abnormal adjacent parenchyma, lobectomy may be preferred over limited resection to ensure complete removal. Finally, adult CPAM resection may be technically challenging because of dense adhesions and distorted tissue planes even in the absence of acute infection, and management is best individualized through multidisciplinary evaluation.
